# Effect of trimetazidine on the functional capacity of ischemic heart disease patients not suitable for revascularization: Meta-analysis of randomized controlled trials

**DOI:** 10.1371/journal.pone.0263932

**Published:** 2022-02-11

**Authors:** Alyaa Ajabnoor, Amnah Mukhtar

**Affiliations:** 1 Faculty of Pharmacy, Department of Pharmacy Practice, King Abdulaziz University, Jeddah, Saudi Arabia; 2 Pharmaceutical Care Division, King Faisal Specialist Hospital and Research Center, Jeddah, Saudi Arabia; Kurume University School of Medicine, JAPAN

## Abstract

**Objective:**

To explore the effect of adding trimetazidine to other anti-anginal drugs on the functional capacity of ischemic heart disease (IHD) patients not suitable for revascularization when compared to first-line antianginal drugs alone.

**Methods:**

MEDLINE and EMBASE databases were searched for English-language peer-reviewed randomized controlled trials (RCTs) comparing trimetazidine with first-line antianginal drugs alone or with placebo in IHD patients not suitable for revascularization and were included in this review. Quality of studies were assessed using the Cochrane collaboration “risk of bias” tool.

**Results:**

Six RCTs, three were crossover studies. A total of 312 participants were included in this review. Overall quality of studies was moderate. Two studies found improvement in the 6-minute walking test (6-MWT) [standardized mean differences (SMD) 1.75; 95% CI 1.35 to 2.14; p <0.001], and two trials found improvement in the Canadian cardiovascular society (CCS) grading of angina class (SMD -1.37; 95% CI -1.89 to -0.84) in the trimetazidine group. Three of the better-quality trials found no increase in total exercise duration (TED) (SMD 0.34; 95% CI -0.10 to 0.78; p < 0.13). Significant heterogeneity was identified among trials describing outcomes for the New York Heart Association (NYHA) functional classification and left ventricular ejection fraction (LVEF %).

**Conclusion:**

Trimetazidine improve walking time and angina severity in IHD patients not suitable for revascularization. Due to the inconsistency of available evidence, RCTs targeting IHD patients with “no option” to undergo coronary revascularization is required to clarify this review question.

## Introduction

Increasing number of patients with coronary artery disease (CAD), are experiencing angina symptoms despite receiving optimal medical therapy. Those patients have limited revascularization options and are frequently referred to as ‘no option’ and have refractory angina (RA) [1]. Estimated annual incidence of RA in Europe is 30–50,000 new cases per year [2]. The European Society of Cardiology (ESC) defined RA as “a chronic condition of ≥3 months, characterized by coronary insufficiency causing angina symptoms in the presence of CAD, that is not amendable to revascularization procedures or medications” [3]. Revascularization procedures; coronary artery bypass graft (CABG) surgery or percutaneous coronary intervention (PCI), are performed to remove the stenosis limiting coronary arteries blood flow. Some patients are not candidate or not planned for revascularization due to history of previous CABG, comorbid conditions, or poor anatomy of coronary arteries [4]. Management of these patients consists of a multidisciplinary approach that shifts the focus of therapy onto symptoms relief and improving quality of life [1].

Trimetazidine, an antianginal drug with metabolic properties that shifts cardiac metabolism from free fatty acids oxidation to glucose oxidation, which consume less oxygen by myocardial cells [5]. This mechanism distinguishes trimetazidine from other conventional antianginal medications; beta-blockers (BBs), calcium channel blockers (CCBs), or nitrates, which exerts cardio depressant activities (decreased heart rate and vasodilation) that limits their efficacy. While trimetazidine relieve angina and enhance exercise tolerance without affecting hemodynamic parameters [3,4]. Trimetazidine is recommended by ESC as add-on therapy to antianginal drugs for treating stable angina in heart failure (HF) patients with reduced ejection fraction, who are symptomatic and intolerant to or inadequately controlled by first line antianginal therapies [6].

Previous systematic reviews evaluated the effect of trimetazidine on exercise tolerance, and left ventricular function of patients with heart HF and ischemic heart disease (IHD) [7–9], found that adding trimetazidine to conventional therapy is effective in improving patients’ symptoms and exercise duration. However, these reviews didn’t focus on IHD patients not suitable for revascularization, therefore, there is no robust evidence for the benefit of using trimetazidine in this subgroup of IHD patients [10]. Therefore, the overreaching aim of this systematic review is to investigate the effect of adding trimetazidine to first-line antianginal drugs on functional and exercise measurements in IHD patients not suitable for revascularization when compared to first-line antianginal drugs alone.

## Methods

The PRISMA guidelines were followed in conducting and reporting the results of this systematic review and meta-analysis (S1 File). There is no previously published review protocol.

### Search strategy

A systematic review and meta-analysis was performed in accordance with the Preferred Reporting Items for Systematic Reviews guidelines [11]. A computer-based systematic search was performed using the Medline database and the Embase database via OVID without date restrictions through December 1^st^, 2020 using a standard form for data extraction. For search terms related to the treatment we used: “trimetazidine,” “vastarel”, “metacard”, or “idaptan”; these terms were all combined with “exercise test”, or “functional capacity”. For disease-related text terms we used “ischemic heart disease”, “heart failure”, “angina”, or “revascularization”. Also, we searched “trimetazidine”, “ischemic heart disease” and “heart failure” as index terms (MeSH) and were exploded as appropriate. The search was limited to clinical trials that involved human subjects of age ≥18 years. The search was restricted to trials published in English language. In addition, a manual search of the references of previous published studies was carried out to identify relevant trials evaluating trimetazidine in IHD patients not eligible for revascularization.

### Study selection

The selection of studies was determined by two reviewers. All identified papers were reviewed according to the following set of inclusion criteria:

1. The study design was Randomized controlled trials (RCTs)–irrespective of blinding.
2. The study population was IHD patients with coronary lesions not suitable for revascularization, patients not candidate or not planned for revascularization.
3. The intervention in the study was oral trimetazidine whether its immediate release (IR), modified release (MR), or sustained release (SR), and combined with first line (conventional) antianginal drugs.
4. Interventions were compared with first-line antianginal drugs alone or with placebo.
5. Study outcomes should include functional capacity measurements; New York Heart Association (NYHA) functional classification, if not reported then exercise total exercise duration (TED) or 6-minute walking test (6-MWT) will be used. Severity of angina assessed using Canadian cardiovascular society (CCS) grading of angina pectoris and echocardiography results of left ventricular ejection fraction (LVEF%).

Studies were excluded according to the following:

1. Study design was not a randomized controlled trial.
2. The study population was non-IHD patients, HF patients from non-ischemic causes, or patients who recently managed with or scheduled to undergo revascularization.
3. The intervention was not the oral form of trimetazidine.
4. The intervention was compared to agents other than first-line antianginal medications (e.g. herbal agents).
5. Trials that did not report any functional capacity measurements.

### Data extraction and quality assessment

Data extraction from studies were performed by two reviewers (AA and AM) using a standardized data extraction form specific for this review topic, where it was customized to obtain information such as; author, country, year of publication, study design, duration, number of patients, dose of trimetazidine, comparator(s), and study outcomes. Any disagreements between authors were resolved by discussion. The quality of the included studies was judged according to the Cochrane risk of bias tool for RCTs, which is a domain-based evaluation tool, that critically assess specific study domains for their risk of bias (low, unclear or high). The methodological quality of each study was assessed for risk of bias using standard criteria: random sequence generation; allocation concealment; blinding of participants, personnel, and outcome assessor; incomplete outcome data; selective reporting; and other potential sources of bias. Crossover trials were evaluated for additional criteria for risk of bias: appropriate crossover design; carry-over effect; and unbiased data [12,13].

### Data synthesis and statistical analysis

The primary outcome was the functional capacity measurements which includes NYHA functional classification, TED or 6-MWT. Secondary outcomes included CCS grading of angina pectoris and LVEF%. Continuous variables were analyzed using the standardized mean differences (SMD) and 95% confidence intervals (CIs). To test for statistical heterogeneity, chi-square-test was set at a significant level of *P*-value <0.1 and *I*^*2*^ of >50%. Moreover, a narrative synthesis was used to complement the quantitative data synthesis by organizing studies into logical categories, describe study findings, and produce a summary of all variations between studies. All measurement data were pooled from included studies and then analyzed for meta-analysis using the computer software package (Review Manager 5.3, 2014).

## Results

### Literature search

**Fig 1:**
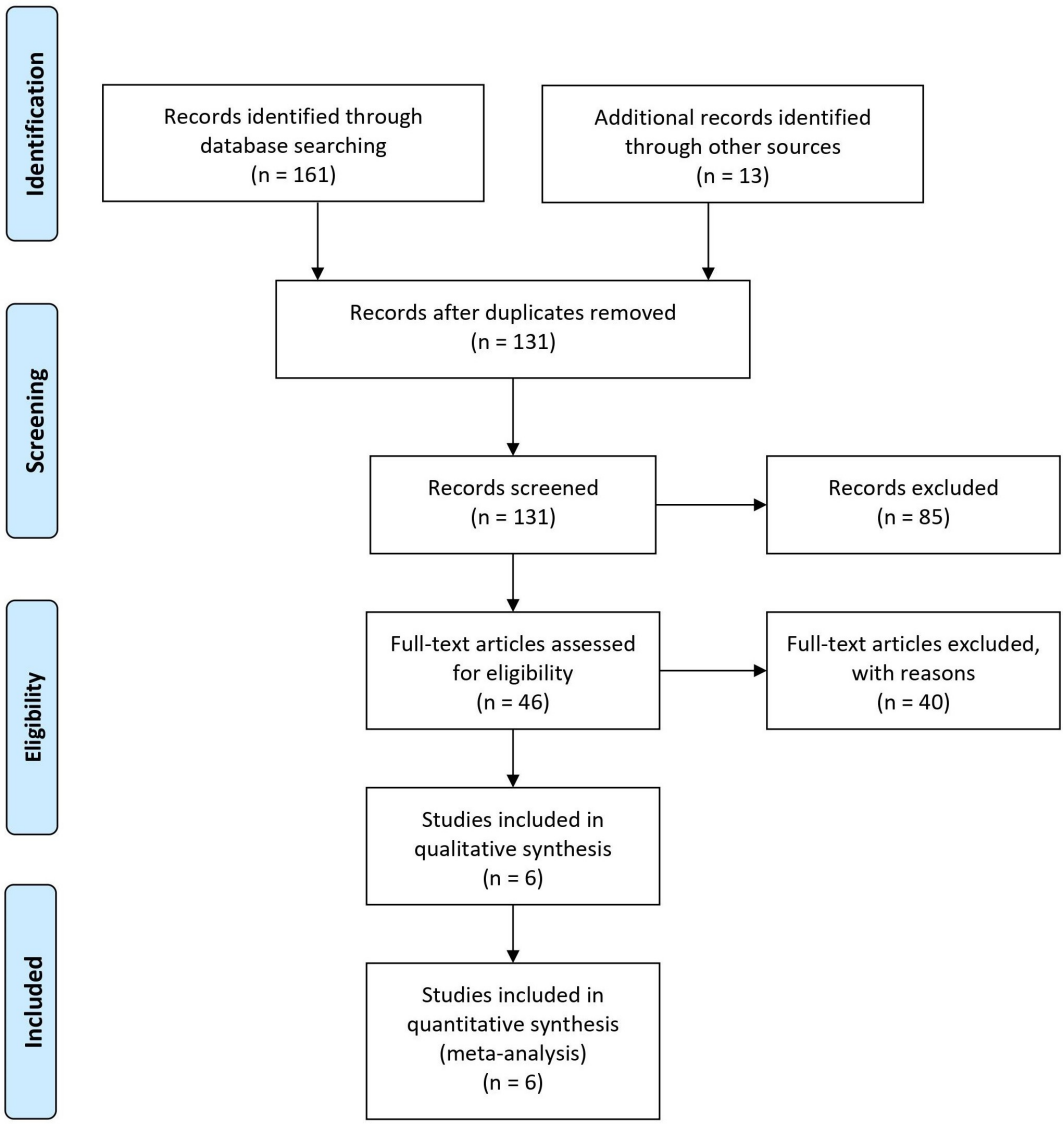
PRISMA 2009 flow diagram demonstrating the search and selection strategy. PRISMA; Preferred Reporting Items for Systematic Reviews and Meta-Analysis.

The electronic searches yielded 161 articles and the manual search from previous meta-analyses and reviews identified an additional 13 articles. Initial screening of the 131 articles comprised examination of title and abstract in the context of our inclusion criteria. Forty-six studies were judged relevant and were further assessed for eligibility. For each study the full paper was read and checked against our selection criteria. Joint decisions on selection were made by the two reviewers, following discussion in case of any discrepancies. Ultimately our systematic review was based on 6 pertinent papers (Fig 1).

### Quality of studies

Across all six included studies, none scored well in all domains of biases, with an overall quality ranging from poor to moderate (Table 1). Only one trial properly reported sequence generation for randomization [14]. Allocation concealment was described as using sealed envelopes by two trials [14,15]. Three trials were high risk for performance bias due to their open label design [14–16], while the rest were low risk as they were described as “double-blinded” [17–19]. The risk of detection bias was low in two trials [22,24], unclear in one [19], and high risk in three trials due to incomplete blinding of outcome assessment [20,21,23] Two trials were high risk for attrition bias due to unequal loss of participants after randomization [19,21], and one trial did not provide information to permit judgment [24]. All six trials were low risk for reporting bias since all pre-specified outcomes were reported across all studies [19–24]. The crossover design was appropriated for the two crossover studies with no biased data. However, both had high risk of bias for not evaluating the carry-over effect [19,22].

**Table 1:** Risk of bias across studies included in the meta-analysis.

Trials	Random sequence generation^$^	Allocation concealment	Participants and personnel blinding	Blinding of outcome assessment	Incomplete outcome data	Selective reporting	Appropriate cross-over design	Carry-over effect	unbiased data	Other bias
**Fragasso et al** [17]§	Unclear risk	Unclear risk	Low risk	Unclear risk	High risk	Low risk	Low risk	Unclear risk	Low risk	Low risk
**Fragasso et al** [14]	Low risk	Low risk	High risk	High risk	Low risk	Low risk	NA	NA	NA	Low risk
**Di Napoli et al** [15]	Unclear risk	Low risk	High risk	High risk	High risk	Low risk	NA	NA	NA	Low risk
**Sisakian et al** [16]	Unclear risk	Unclear risk	High risk	High risk	Low risk	Low Risk	NA	NA	NA	Low risk
**Ribeiro et al** [18]§	Unclear risk	Unclear risk	Low risk	Low risk	Low risk	Low risk	Low risk	Unclear risk	Low risk	Low risk
**Momen et al** [19]	Unclear risk	Unclear risk	Low risk	Low risk	Unclear risk	Low risk	NA	NA	NA	Low risk

^§^In case of crossover studies, it refers to randomization of treatment order. NA, not applicable.

### Study characteristics

The characteristics of the six included studies are summarized in Table 2. The six included studies were RCTs, two were crossover trials [14–19], three were open-label [14–16], and the rest were double-blinded placebo-controlled trials [17–19]. Studies were conducted from 2003 to 2016. One trial studied the effect of trimetazidine over a period of 48 months [15], while other trials lasted for 3 to 12 months [14,16–19]. Trimetazidine dose ranged from 60 to 70 mg/day. Most trials used IR form of trimetazidine, except for two studies in which MR/SR formulations were used [16,19]. A total of 312 participants were included from all studies, average age was 60s (Table 3) [14–19]. One trial included subgroup analysis of ischemic and non-ischemic HF patients, of which only the subgroup of patients with IHD were included in this review [14]. Men comprised a higher proportion of participants in most studies [15–19]. In five trials patients had LVEF <45%, except for one trial that included patients with LVEF >45% [18]. Of the four studies describing diabetes status of patients [15,17–19], approximately 100 patients were diabetics. All participants were IHD patients receiving a combination of standard anti-ischemic treatment, including first line anti-anginal drugs. From the studies describing the antianginal medications of patients, it was estimated that 51% of patients were on β-Blockers, and 67% were on nitrates.

**Table 2:** Characteristics of included studies.

Trials	Publication year	Duration of study	Location	Study design	Number of enrolled patients (TMZ/control)	Patients age (years)	Intervention	Comparator	Characteristics of included patients	Study outcomes
**Fragasso et al** [17]	2003	6 months	Single site in Italy	Double blind cross-over	16/16	64 (mean)	TMZ 60mg/day + conventional therapy	Placebo + Conventional therapy	IHD, DM, LVEF ≤45%, persistent symptoms, undergone maximal attainable revascularization or not amendable to revascularization	TED LVEF%
**Fragasso et al** [14]	2006	12 months	Single site in Italy	Open-label RCT	18/17	65 (mean)	TMZ 60mg/day + conventional therapy	Conventional therapy	IHD, persistent symptoms, LVEF ≤45%, with no coronary lesions suitable for revascularization	TED LVEF%
**Di Napoli et al** [15]	2007	48 months	Single site in Italy	Open-label RCT	30/31	67 (mean)	TMZ 60mg/day + conventional therapy	Conventional therapy	IHD, previous MI, LVEF <40%, NYHA II-IV, with coronary lesions unsuitable for revascularization	NYHA 6-MWT LVEF%
**Sisakian et al** [16]	2007	3 months	Single site in Armenia	Open-label RCT	42/40	63.5 (mean)	MR TMZ 70mg/day + conventional therapy	Conventional therapy	IHD, previous MI, LVEF <40%, NYHA II-III, with coronary lesions unsuitable for revascularization	NYHA 6-MWT LVEF%
**Ribeiro et al** [18]	2007	6 weeks	Single site in Brazil	Double blind cross-over	10/10	62 (mean)	TMZ 60mg/day + conventional therapy	Placebo + Conventional therapy	IHD, type 2 diabetes, CCS class II-III, on full-anti-ischemic treatment, not eligible for revascularization	TED CCS class
**Momen et al** [19]	2016	6 months	Single site in Bangladesh	Double blind parallel RCT	55/53	58.5 (mean)	SR TMZ 35mg BID + Conventional therapy	Placebo + Conventional therapy	Documented CAD, history of MI, decompensated HF, LVEF ≤40%, NYHA I-III, not planned for revascularization	NYHA CCS class LVEF%

MR, Modified Release; SR, Sustained Release; BID, twice a day; LVEF, Left ventricular ejection fraction; MI, Myocardial infarction; TED, total exercise duration; 6-MWT, 6 minutes walking test.

**Table 3:** Patient characteristics.

Trials	Patients N (TMZ/Control)	Age (years) (TMZ/Control)	Male gender N (TMZ/Control)	LVEF% (TMZ/Control)	Diabetic patients N (TMZ/Control)	Patients on BBs (TMZ/Control)	Patients on CCBs (TMZ/Control)	Patients on Nitrate therapy (TMZ/Control)
**Fragasso et al** [17]*	16/16	64±7	16/16	40%±5%/40%±5%	16/16	10/10	1 /1	9/9
**Fragasso et al** [14]§	18/17	NA	NA	36%±6%/39%±5%	NA	NA	NA	NA
**Di Napoli et al** [15]	30/31	67±5.5/69±7	17/18	30%±6%/31%±8%	10/11	15/18	6/6	6/8
**Sisakian et al** [16]	42/40	63.5±9.3/65.6±8.7	37/33	34.5%±3.8%/32.4%±5.6%	NA	38/40	5/3	19/20
**Ribeiro et al** [18]*	10/10	62±8	6/6	56%±9%/56%±9%	10/10	NA	NA	NA
**Momen et al** [19]	55/53	58±9.5/59±8.9	45/41	32.9%±6.6%/33.1%±6.2%	26/27	42/43	0	34/35

*Cross-over studies.

^§^Only subgroup of patients with IHD were included in this review. NA, not available.

### Primary outcome: Functional capacity

**Fig 2:**
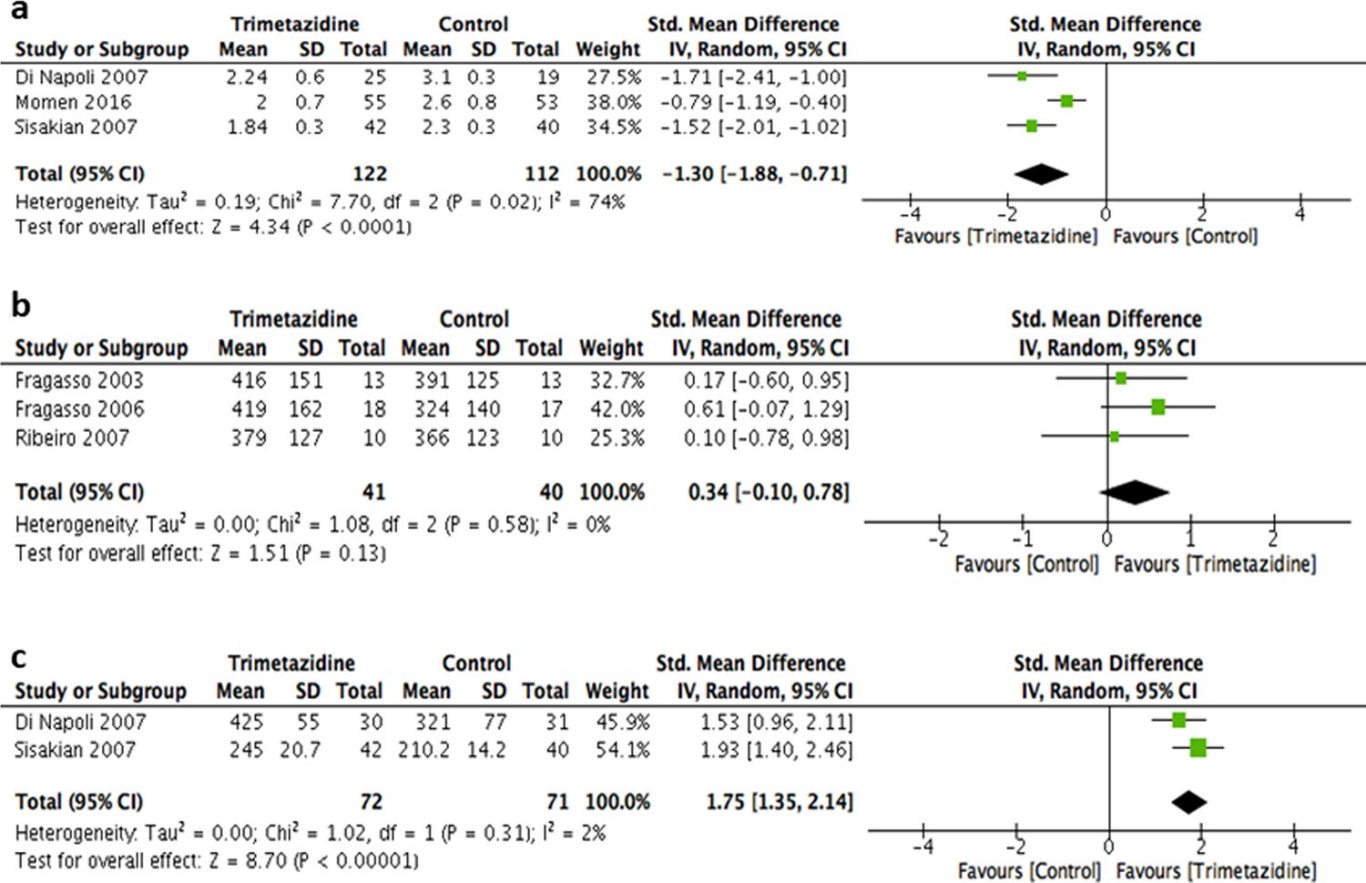
Forest plots showing the SMDs for the parameters of patient’s functional capacity: **2a)** New York heart association (NYHA) functional classification; **2b)** total exercise duration (TED); **2c)** six-minute walk test (6-MWT). *CI* confidence interval.

The change in NYHA functional classification was reported in 3 of the included studies (Fig 2). All 3 studies reported improvement in mean NYHA classification after the intervention period [15,16,19]. A statistically significant improvement in NYHA was observed with trimetazidine (SMD -1.30; 95% CI -1.88 to -0.71; p <0.01), but with significant heterogeneity (*P* = 0.02, *I*^*2*^ = 74%) in fixed effect and random effect models (Fig 2). From the 3 trials reporting data on exercise capacity, TED was not significantly increased after receiving trimetazidine compared with controlled patients (SMD 0.34; 95% CI -0.10 to 0.78; *P* < 0.13), with no evidence of heterogeneity (p = 0.58, *I2* = 0%) [14,17,18]. Additionally, walking test in meters (6-MWT) was reported in 2 trials and it was found that trimetazidine significantly increased walking distance compared with the controlled patients (SMD 1.75; 95% CI 1.35 to 2.14; *P* <0.001), with no evidence of heterogeneity (p = 0.31, *I2* = 2%) (Fig 2) [15,16].

### Secondary outcomes: CCS angina class and LVEF

**Fig 3:**
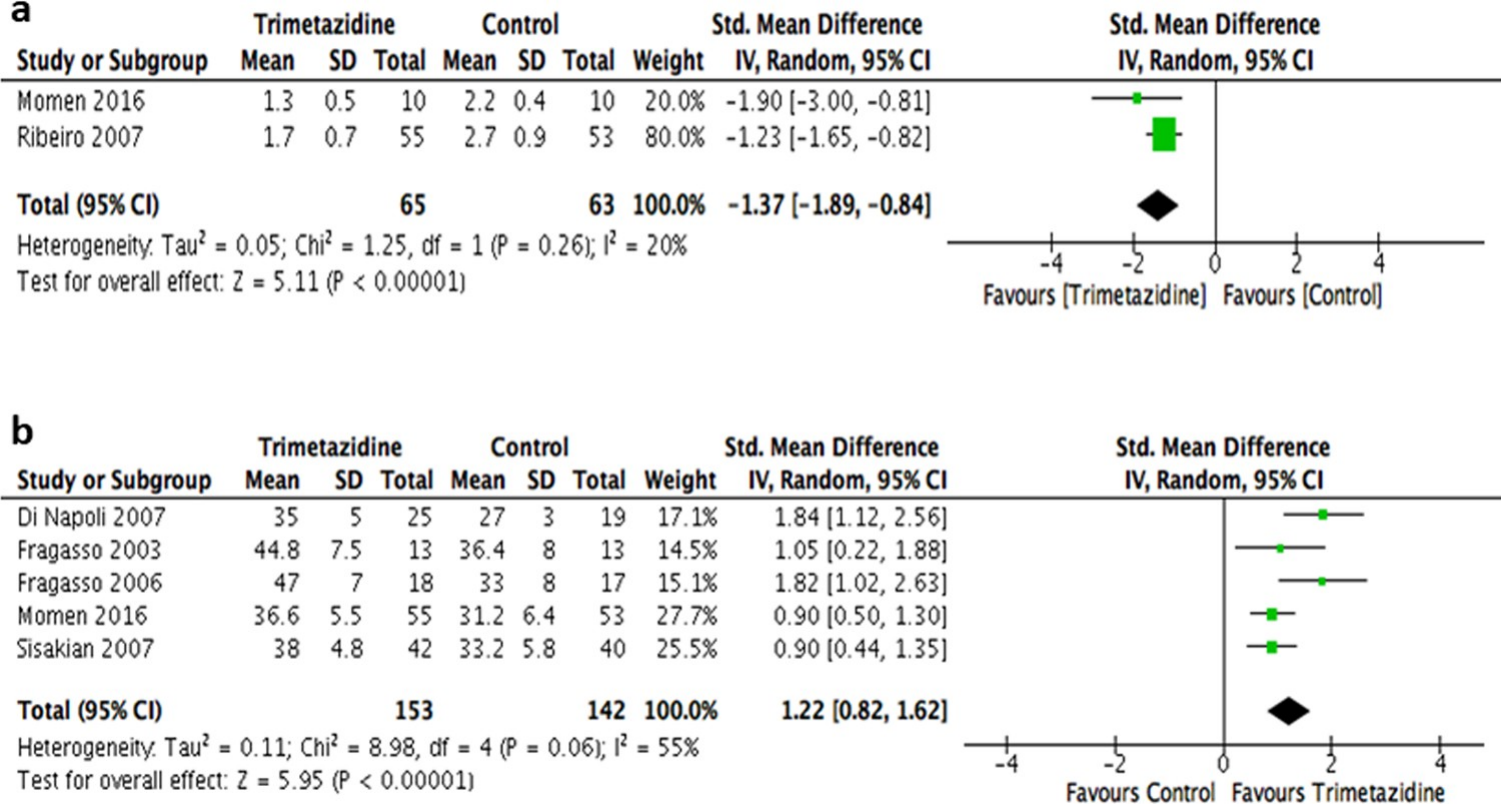
Forest plots showing the SMDs for the secondary parameters of: **3a)** Canadian cardiovascular society (CCS) angina class; **3b)** left ventricular ejection fraction (LVEF).

Secondary outcomes were analyzed using fixed-effect model in which a statistically significant differences were observed with both outcomes. The CCS angina class was improved in the trimetazidine group compared to controlled patients (SMD -1.37; 95% CI -1.89 to -0.84), with no significant heterogeneity (*P* = 0.26, *I2* = 20%). Also, an improvement in LVEF% was observed with trimetazidine as reported in 5 of the included studies (SMD 1.22; 95% CI 0.82 to 1.62), but with evidence of heterogeneity (*P* = 0.06, *I2* = 55%) (Fig 3).

### Publication bias

Funnel plot was constructed using the SMD values obtained from the functional capacity measurements (S1 File). The shapes of the funnel seemed asymmetrical, suggesting that publication bias in the analysis could not be ruled out. However, given the small number of included studies in this review the power of the test was too low to distinguish chance from real asymmetry.

### Sensitivity analysis

There was no difference in the results between the fixed effect model and the random-effect model for all outcomes. Sensitivity analysis was conducted on outcomes generated from meta-analysis of more than two studies. We repeated the analysis for methodological quality (excluding cross-over studies) and found that there was still a significant improvement in NYHA and LVEF% with TMZ treatment compared with conventional therapy. In addition, for NYHA and LVEF% removing any study had no effect on the pooled SMD and no study crossed zero. The study conducted by Momen et al, [19] was the major contributor to heterogeneity test in the analysis for NYHA, while studies by Fragasso et al, [14] and Di Napoli et al, [15] have contributed to the heterogeneity in the analysis for LVEF%; however, no study affected the findings of statistical significance in favour of TMZ.

## Discussion

In this study we performed a meta-analysis of trials that evaluated the effect of combining TMZ with conventional antianginal drugs on the functional capacity of IHD patients not suitable for revascularization. We have found an overall improvement with TMZ in the functional capacity measurements mainly for NYHA functional classification and 6-MWT. We also assessed the effect of TMZ on CCS angina class and LVEF%. The results suggest that TMZ could improve patient’s angina symptoms and cardiac function more effectively in the setting of decreased LVEF%.

While most conventional antianginal drugs exhibit their effect through hemodynamic function to balance myocardial oxygen supply and demand, trimetazidine protect cardiac myocytes from ischemia through the inhibition of fatty acid metabolism and to a lesser extent by stimulating glucose metabolism. In theory, this protective mechanism limits the loss of myocytes due to ischemia in patients with angina which ultimately improve their prognosis [20].

Earlier clinical trials have evaluated the efficacy of trimetazidine monotherapy in stable angina patients with ischemic cardiomyopathy and in acute MI [21], or as combined therapy with other conventional antianginal drugs in stable angina patients [22–24]. According to a previous meta-analysis of 13 clinical trial that consisted of 1628 patients have found that adding trimetazidine to conventional antianginal drugs was associated with fewer weakly angina attacks, less use of nitroglycerin, higher total work, and prolonged exercise duration at peak exercise compared to other antianginal agents [7]. These findings combined support trimetazidine use and as recommended by the 2019 recent ESC guidelines, trimetazidine can be prescribed in two clinical scenarios; 1) as a second-line treatment in subjects who cannot tolerate or have contraindications to, or their symptoms not controlled by BBs, CCBs, and nitrates; 2) as a first-line treatment with combination with BBs or CCBs in selected patients according to their heart rate, blood pressure, and tolerance [25].

Care for patients with refractory angina and who are not suitable for revascularization could be challenging and different across the world due to the limited guidance available from national practice guidelines, and the lack of standardized definitions to address and identify this complex group of patients. This emphasize the need for robust RCTs focusing on patients with refractory angina before trimetazidine can be definitely recommended in this group of patients [1]. While most of the available trials on trimetazidine focused on evaluating its effect on IHD patients, in this meta-analysis we managed to identify the few studies that evaluated trimetazidine effect in patients with refractory angina and with no option for revascularization.

In a meta-analysis performed by Gao et al, seven of the included studies reported data on NYHA classification and have found a significant improvement in NYHA classification compared to conventional therapy [9]. In our meta-analysis three studies have found that NYHA classification was improved by the addition of trimetazidine to standard antianginal drugs, however, these findings were from moderate quality studies with significant heterogeneity that may be attributed to differences in baseline NYHA classification and cardiac function [15,16,19]. Compared to the meta-analysis results by Zhao et al, which found a significant improvement in TED and the 6-MWT with trimetazidine in IHD patients [26], our finding were similar with regard to the 6-MWT but not for the TED as trimetazidine did not result in significant improvement compared to conventional drugs. This might be due to the different characteristics of IHD patients included in the meta-analysis by Zhao et al, compared to our meta-analysis since we focused on patients not suitable for revascularization and the fact that the two of the studies that evaluated trimetazidine effect on TED were cross-over in design which could represent a great methodological difference. Additionally, consistent with a previous meta-analysis We have also found a significant improvement in LVEF% in five studies after adding trimetazidine to standard antianginal drugs [14–17,19]. However, the meta-analysis found significant heterogeneity among these studies, which could be attributed to studies including lower or higher LVEF% than other studies.

In order to extract evidence to recommend adding trimetazidine to conventional therapy for this specific group of IHD patients, results from this meta-analysis should be interpreted with caution due to some limitations. First, the methodological quality of included studies was less than optimal, with three open-label studies in which performance bias from these trials could not be excluded, as well as two trials being high risk for attrition bias. One reason for including studies of lower than optimal quality could be the high risk population that we addressed in this review, since we focused only on patients with “no option” to undergo revascularization and therefore our search strategy have captured only small cross-over trials and open-label RCTs that were conducted to assess the effect of TMZ in this high-risk group. Second, the number of patients included in this meta-analysis is small, making estimates of functional capacity based on small number of measures. Although, it is acceptable to perform a meta-analysis on a minimum of two studies, estimation of intervention effect would be more reliable if more information were included [13]. Third, due to the limited number of included studies evaluating the effect of trimetazidine across different sub-group of patients such as diabetics, or according to the duration of trimetazidine use was not feasible.

In conclusion, this meta-analysis shows that trimetazidine significantly improves walking time and angina severity in IHD patients not suitable for revascularization. Trimetazidine effects may also be associated with positive changes in NYHA classification and LVEF%. Given these results, large-scale RCTs targeting IHD patients with “no option” to undergo revascularization is required to clarify this review question. Trimetazidine might be an option for this group of patients, however, until now there is no clear evidence to support it routine use as a second-line agent.

## Supporting information

S1 Checklist PRISMA 2009 checklist. (DOC)

S1 File Funnel plots. (DOCX)

## References

[1] Henry TD, Satran D, Jolicoeur EM. Treatment of refractory angina in patients not suitable for revascularization. Nat Rev Cardiol. 2014;11:78–95. doi: 10.1038/nrcardio.2013.200 24366073

[2] Sainsbury PA, Fisher M, De Silva R. Alternative interventions for refractory angina. Heart. 2017;103(23):1911–22. doi: 10.1136/heartjnl-2015-308564 28954830

[3] Mannheimer C, Camici P, Chester MR, Collins A, DeJongste M, Eliasson T, et al. The problem of chronic refractory angina: Report from the ESC Joint Study Group on the treatment of refractory angina. Eur Heart J. 2002;23:355–70. doi: 10.1053/euhj.2001.2706 11846493

[4] Thadani U. The pursuit of optimum outcomes in stable angina. Am J Cardiovasc Drugs. 2003;3:11–2.

[5] Ferrari R, Ford I, Fox K, Marzilli M, Tendera M, Widimský P, et al. A randomized, double-blind, placebo-controlled trial to assess the efficAcy and safety of Trimetazidine in patients with angina pectoris having been treated by percutaneous coronary intervention (ATPCI study): Rationale, design, and baseline characteristi. Am Heart J. 2019;210:98–107. doi: 10.1016/j.ahj.2018.12.015 30771737

[6] Ponikowski P, Voors A, Anker SD, Bueno H, Cleland JGF, Coats AJS, et al. 2016 ESC Guidelines for the diagnosis and treatment of acute and chronic heart failure. Eur Heart J. 2016;27:2129–200. 10.1093/eurheartj/ehw128 27206819

[7] Peng S, Zhao M, Wan J, Fang Q, Fang D, Li K. The efficacy of trimetazidine on stable angina pectoris: A meta-analysis of randomized clinical trials. Int J Cardiol. 2014;177:780–5. doi: 10.1016/j.ijcard.2014.10.149 25466565

[8] Zhao Y, Peng L, Luo Y, Li S, Zheng Z, Dong R, et al. Trimetazidine improves exercise tolerance in patients with ischemic heart disease: A meta-analysis. Verbesserung der Belastungstoleranz durch Trimetazidin bei Patienten mit ischamischer Herzerkrankung Eine Metaanalyse. 2016;41:514–22. doi: 10.1007/s00059-015-4392-2 26668006

[9] Gao D, Ning N, Niu X, Hao G, Meng Z. Trimetazidine: A meta-analysis of randomised controlled trials in heart failure. Heart. 2011;97:278–86. doi: 10.1136/hrt.2010.208751 21134903

[10] McGillion M, Arthur HM, Cook A, Carroll SL, Victor JC, L’Allier PL, et al. Management of Patients With Refractory Angina: Canadian Cardiovascular Society/Canadian Pain Society Joint Guidelines. Can J Cardiol. 2012;28:S20–41. doi: 10.1016/j.cjca.2011.07.007 22424281

[11] Moher D, Liberati A, Tetzlaff J, Altman DG, Altman D, Antes G, et al. Preferred reporting items for systematic reviews and meta-analyses: The PRISMA statement. Ann Intern Med. 2009;151(4):264–9. doi: 10.7326/0003-4819-151-4-200908180-00135 19622511

[12] Ding H, Hu GL, Zheng XY, Chen Q, Threapleton DE, Zhou ZH. The method quality of cross-over studies involved in Cochrane Systematic Reviews. PLoS One. 2015;10(4):1–8. doi: 10.1371/journal.pone.0120519 25867772 PMC4395015

[13] Higgins J, Green S, Eds. Chapter 23: Including variants on randomized trials. In: Cochrane handbook for systematic reviews of interventions Version 510. 2011.

[14] Fragasso G, Palloshi A, Puccetti P, Silipigni C, Rossodivita A, Pala M, et al. A Randomized Clinical Trial of Trimetazidine, a Partial Free Fatty Acid Oxidation Inhibitor, in Patients With Heart Failure. J Am Coll Cardiol. 2006;48(5):992–8. doi: 10.1016/j.jacc.2006.03.060 16949492

[15] Napoli P Di, Giovanni P Di, Gaeta MA, Taccardi AA, Barsotti A. Trimetazidine and Reduction in Mortality and Hospitalization in Patients With Ischemic Dilated Cardiomyopathy: A Post Hoc Analysis of the Villa Pini DʼAbruzzo Trimetazidine Trial. J Cardiovasc Pharmacol. 2007;50(5):585–9. doi: 10.1097/FJC.0b013e31814fa9cb 18030070

[16] Sisakian H, Torgomyan A, Barkhudaryan A. The effect of trimetazidine on left ventricular systolic function and physical tolerance in patients with ischaemic cardiomyopathy. Acta Cardiol. 2007;62(5):493–9. doi: 10.2143/AC.62.5.2023413 17982971

[17] Fragasso G, Piatti MD P., Monti L, Palloshi A, Setola E, Puccetti P, et al. Short- and long-term beneficial effects of trimetazidine in patients with diabetes and ischemic cardiomyopathy. Am Heart J. 2003;146(5):854. doi: 10.1016/S0002-8703(03)00415-0 14597947

[18] Ribeiro LW, Ribeiro JP, Stein R, Leitão C, Polanczyk CA. Trimetazidine added to combined hemodynamic antianginal therapy in patients with type 2 diabetes: a randomized crossover trial. Am Heart J. 2007;154(1):78.e1–78.e7. doi: 10.1016/j.ahj.2007.04.026 17584555

[19] Ali MZ, Khalil MI, Khan MR, Ali M, Goni MN, Karmakar PK, et al. Effects of sustained-release trimetazidine on chronically dysfunctional myocardium of ischemic dilated cardiomyopathy–Six months follow-up result. Indian Heart J. 2016;68(6):809–15. doi: 10.1016/j.ihj.2016.03.021 27931552 PMC5143824

[20] Giannopoulos AA, Giannoglou GD, Chatzizisis YS. Pharmacological approaches of refractory angina. Pharmacol Ther. 2016;163:118–31. doi: 10.1016/j.pharmthera.2016.03.008 27013345

[21] Labrou A, Giannoglou G, Zioutas D, Fragakis N, Katsaris G, Louridas G. Trimetazidine administration minimizes myocardial damage and improves left ventricular function after percutaneous coronary intervention. Am J Cardiovasc Drugs. 2007;7:143–50. doi: 10.2165/00129784-200707020-00006 17503885

[22] Szwed H, Hradec J, Préda I. Anti-ischaemic efficacy and tolerability of trimetazidine administered to patients with angina pectoris: Results of three studies. Coron Artery Dis. 2001;12(Suppl 1):S25–8. 11286304

[23] Kölbel F, Bada V. Trimetazidine in geriatric patients with stable angina pectoris: The tiger study. Int J Clin Pract. 2003;57(10):867–70. 14712887

[24] Chazov EI, Lepakchin VK, Zharova EA, Fitilev SB, Levin AM, Rumiantzeva EG, et al. Trimetazidine in angina combination therapy—The TACT study: Trimetazidine versus conventional treatment in patients with stable angina pectoris in a randomized, placebo-controlled, multicenter study. Am J Ther. 2005;12:35–42. doi: 10.1097/00045391-200501000-00006 15662290

[25] Knuuti J, Wijns W, Achenbach S, Agewall S, Barbato E, Bax JJ, et al. 2019 ESC guidelines for the diagnosis and management of chronic coronary syndromes. Eur Heart J. 2019;00:1–71. 10.1093/eurheartj/ehz425 31504439

[26] Zhao Y, Peng L, Luo Y, Li S, Zheng Z, Dong R, et al. Trimetazidine improves exercise tolerance in patients with ischemic heart disease: A meta-analysis. Herz. 2016;41:514–22. doi: 10.1007/s00059-015-4392-2 26668006

