# Supplementary material for: Effect of trimetazidine on the functional capacity of ischemic heart disease patients not suitable for revascularization: Meta-analysis of randomized controlled trials
Source: PLoS One. 2022 Feb 11;17(2):e0263932. doi: 10.1371/journal.pone.0263932 (PMC8836318; doi:10.1371/journal.pone.0263932)

**Figure S1. Funnel Plot of Included Studies for total exercise duration (TED)**


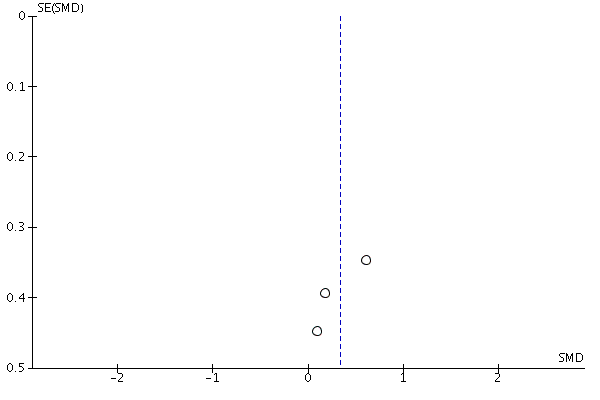


**Figure S2. Funnel Plot of Included Studies for New York Heart Association (NYHA)**


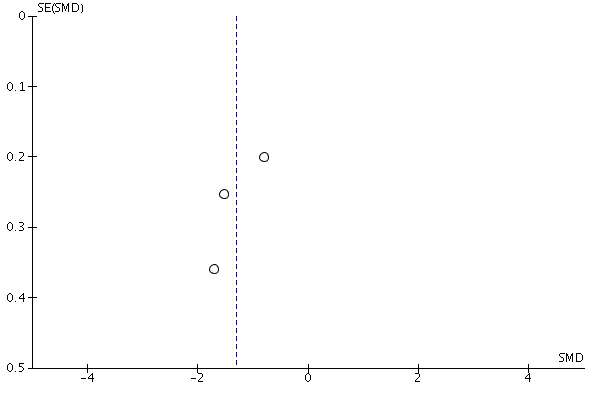


**Figure S3. Funnel Plot of Included Studies for left ventricular ejection fraction (LVEF%)**


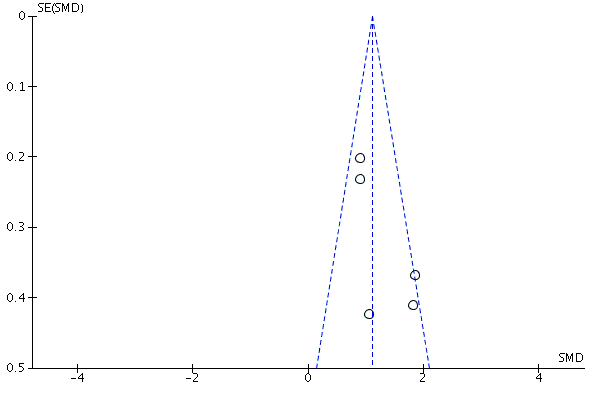

Supplement: S1 File — (DOCX) [file pone.0263932.s002.docx]
